# Targeted Photodynamic Diagnosis and Therapy for Esophageal Cancer: Potential Role of Functionalized Nanomedicine

**DOI:** 10.3390/pharmaceutics13111943

**Published:** 2021-11-16

**Authors:** Onyisi Christiana Didamson, Heidi Abrahamse

**Affiliations:** Laser Research Centre, Faculty of Health Sciences, University of Johannesburg, P.O. Box 17011, Doornfontein, Johannesburg 2028, South Africa; christydidamson@gmail.com

**Keywords:** esophageal cancer, photodynamic diagnosis (PDD), photodynamic therapy (PDT), photosensitizer (PS), functionalized nanomedicine, nanoparticles (NP)

## Abstract

Esophageal cancer is often diagnosed at the late stage when cancer has already spread and is characterized by a poor prognosis. Therefore, early diagnosis is vital for a better and efficient treatment outcome. Upper endoscopy with biopsy is the standard diagnostic tool for esophageal cancer but is challenging to diagnose at its premalignant stage, while conventional treatments such as surgery, chemotherapy, and irradiation therapy, are challenging to eliminate the tumor. Photodynamic diagnosis (PDD) and therapy (PDT) modalities that employ photosensitizers (PSs) are emerging diagnostic and therapeutic strategies for esophageal cancer. However, some flaws associated with the classic PSs have limited their clinical applications. Functionalized nanomedicine has emerged as a potential drug delivery system to enhance PS drug biodistribution and cellular internalization. The conjugation of PSs with functionalized nanomedicine enables increased localization within esophageal cancer cells due to improved solubility and stability in blood circulation. This review highlights PS drugs used for PDD and PDT for esophageal cancer. In addition, it focuses on the various functionalized nanomedicine explored for esophageal cancer and their role in targeted PDD and PDT for diagnosis and treatment.

## 1. Introduction

Esophageal cancer is ranked as the eighth most lethal neoplastic disease by incidence and the sixth cause of cancer-associated death globally, with a 5-year survival rate lower than 20%. Furthermore, a recent report from the Global Cancer Observatory reveals that in the year 2020, esophageal cancer accounts for 604,100 (3.1%) new cases and 544,076 (5.5%) deaths of all cancer incidences worldwide, and its prevalence is aggressively increasing [1]. Esophageal cancer emerges from the continuous insults of the esophageal mucosa resulting in cellular and molecular alterations in the mucosa, consequently leading to the neoplastic phenotype.

Histologically, esophageal cancer can be categorized into two types: esophageal squamous cell carcinoma (OSCC) and esophageal adenocarcinoma (OAC) [2]. These two cancer types are epidemiologically and histologically different. Esophageal squamous cell carcinoma is the most prevalent type of esophageal cancer, and it represents a 90% incidence of esophageal cancer worldwide. This cancer type has a high incidence rate in Asia, Africa, and South America. The main predisposing factors for OSCC include smoking, alcohol, poor oral hygiene, ingestion of causative substances, and lack of balanced diets. Esophageal adenocarcinoma is mostly found in developed countries compared to developing countries. The main predisposing factors are age, gastresophageal reflux disease (GORD), smoking, obesity, and diet deficiency in fruits/vegetables [3,4].

## 2. Classic Diagnosis of Esophageal Cancer

Conventional diagnostic tests of esophageal cancer consist of physical/medical examination and imaging along with biopsy [5]. A physical and medical examination is the first diagnostic procedure, and these take into account the clinical presentations associated with esophageal cancer such as nausea or vomiting with weight loss, reflux, swallowing difficulty, or upper abdominal pain, as well as the risk factors associated with esophageal cancer [6]. Imaging diagnostic procedures are performed following cancer suspicions, and these involve examining the esophagus walls to detect alterations, irritations, and the extend of metastases. These procedures include barium esophagram, endoscopy, bronchoscopy, endoscopic ultrasound (EUS), computerized tomography (CT), magnetic resonance imaging (MRI), and fluorodeoxyglucose positron emission tomography (FDG-PET) [7]. A biopsy is a vital procedure that helps characterize the histological cell types of esophageal cancer and provide more cellular and molecular information of the cancer cells [8]. Significant advancements have been made in conventional diagnostic procedures for esophageal cancer. However, these approaches are unable to identify preneoplastic lesions at their earliest stage. A devasting challenge with esophageal cancer is the late presentation of symptoms, leading to delayed diagnosis, poor prognosis, advanced tumor stages, and high mortality rates.

## 3. Classic Treatment of Esophageal Cancer

The standard treatment for esophageal cancer is a multimodal approach which includes chemotherapy, chemoradiotherapy, and surgical resection or combinations of these therapies. The treatment of choice may be curative or palliative, depending on the type, size, location, and stage of cancer [9,10].

Surgery is commonly used as a curative treatment regimen for esophageal cancer to eradicate the tumor and stop its growth and spread. The surgical approach applied could be endoscopic resection or esophagectomy (surgical resection), depending on the tumor location and extent of spread. Neoadjuvant treatment, which consists of either chemotherapy or radiation therapy and in combination, may be administered before surgery to shrink the tumor. Furthermore, chemotherapy and/or radiation therapy may also be given post-surgery as an adjuvant treatment to eradicate any tumor cell remnants. Surgical therapies are mainly recommended for early-stage cancer. Despite being the most commonly employed treatment, it is without doubt associated with complications which include sepsis, hemorrhage, blood clots, damage to adjacent organs, stricture of the esophagus from surgical wounds, and collapse of the vocal cords [7,11].

Radiation therapy involves applying radioactive substances and X-rays, targeting cancer cells to remove and hurt their proliferation. This treatment could be used in the following scenario, (a) alone as a curative therapy to kill cancer cells in cases where surgery cannot be conducted, (b) as neoadjuvant therapy alone or in combination with chemotherapy before surgery, to reduce the tumor size (c) as adjuvant therapy alone or in combination with chemotherapy after surgery, to eliminate any leftover cancer, and (d) as palliative therapy with the aims to enhance the quality of life to alleviate the pains and symptoms. The disadvantage of radiation is that it results in genetic alteration of adjacent non-cancerous tissue cells, fatigue, skin irritations, loss of appetite, and weight loss [7,9].

Chemotherapy represents the use of anticancer agents administered systemically to destroy cancer cells and limit tumor growth. Chemotherapy can be used as curative, neoadjuvant, adjuvant, and palliative therapy. Palliative chemotherapy is often administered to patients with advanced-stage cancer. The standard chemotherapy agents for esophageal cancer include cisplatin, 5-fluorouracil, doxorubicin, capecitabine, docetaxel, irinotecan, oxaliplatin, paclitaxel, trifluridine-tripiracil, entrectinib, labrotrectinib, capecitabine, carboplatin, and leucovorin [12,13]. These drugs are usually given in combinations to improve their efficacies. Furthermore, to limit off-target, increase optimal therapeutic effects, and improve survival outcomes, chemotherapeutic agents are now administrated with some molecular target drugs such as trastuzumab, nivolumab, pembrolizumab, ramucirumab, fam-trastuzumab deruxtecan-nxki, and cetuximab [12,14,15]. Early-stage cancer treated with chemotherapy has shown improved survival rates, particularly in combination with radiation, while a poor prognosis is observed in advanced-stage esophageal cancer [15,16]. The continuous usage of the chemotherapeutic agent is characterized by dose-limiting cytotoxicity; drug resistance; and other adverse effects such as neuropathy, neutropenia, pneumonitis, bradycardia myelosuppression, thereby limiting the extensive use of these agents [13].

Chemoradiotherapy (CRT) is the combination of chemo and radiotherapy and the standard gold treatment for advanced-stage esophageal cancer in the absence of distant metastasis. Generally, CRT is administered to individuals who opt-out or are unfit for surgery. The outcome of CRT depends on the stage of cancer, and individuals who attained a favorable outcome often have cancer relapse. Therefore, salvage therapy is conducted to eliminate remnant cancer tissue or tumor relapse [17]. Among the several salvage therapies available, photodynamic therapy, which will be discussed in detail below, has shown improved outcome, especially for stage T1a and T1-2 remnant cancer tissue or cancer relapse. Studies on PDT as salvage therapy for local failure following CRT on esophageal cancer have shown significant treatment improvements and survival outcomes. These studies reported complete response rates of 98.3% [18], 83.3% [19], and 58.4% [20]. Furthermore, the overall survival rates were 41.1% (5-year) and 80.0% (2-year) survival rates, respectively. Though PDT is associated with esophageal fistula and stricture, these findings show that PDT is a promising treatment alternative when cancer relapse has not extended beyond the T1-2 stage.

The standard treatment options have contributed to significant progress in esophageal cancer treatment. However, they are still faced with setbacks such as suboptimal therapy, adverse side effects, chemoresistance, disease recurrence and a 5-year survival rate of less than 20%, thereby posing a severe public health threat. Therefore, the need to investigate an alternative diagnostic and treatment option that can detect esophageal cancer at its premalignant stage, efficiently eliminate cancer cells, boost patient survival rate and limit metastasis while limiting side effects remain essential.

## 4. Photodynamic Diagnosis (PDD) and Therapy (PDT) for Esophageal Cancer

The conventional diagnosis and treatments for esophageal cancer are limited due to adverse side effects, invasiveness, and inefficiency in eliminating its advanced stage. Furthermore, early esophageal cancer cannot be detected with the present white light endoscopy. Therefore, the quest for alternative diagnostic and treatment options are necessary. The key to optimum prognosis of cancer is early detection, which prompt early treatment, improves the survival rate, and limits the spread [21]. Photodynamic diagnosis (PDD) and therapy (PDT) are emerging modalities for cancer detection and treatment.

Photodynamic diagnosis (PDD) is a diagnostic measure that employs a photosensitizer to detect cancerous cells. When exposed to visible light, results in the emission of fluorescence that facilitates early detection of the preneoplastic lesion. Photosensitizers used in PDD are characterized by their affinity for neoplastic tissues, low toxicity, and emitting fluorescence within the lower visible blue light spectrum of wavelength. The application of PDD can allow easy visualization and identification of cancer cells, thereby facilitating prompt diagnosis and optimal prognosis [22]. The conventional photosensitizer used in PDD of esophageal cancer includes 5-aminolevulinic acid (5-ALA), indocyanine green, and methylene blue [23,24,25].

Photodynamic therapy is a noninvasive treatment that involves applying a photosensitizer activated at a particular wavelength with the production of reactive oxygen species that result in cell death. It is characterized by minimal side effects compared to conventional treatments. PDT offers a safe and efficient approach that discriminatively eliminate tumor cells from normal tissues. This treatment option enhances target tumor specificity and substantially minimizes side effects than the standard chemotherapy and radiotherapy as only the diseased tissue is exposed to treatment. Furthermore, PDT is most suitable in patients where the tumor location or size restricts standard therapy application [26,27]. As PDD and PDT employ the same theranostic mechanism that requires the application of photosensitizers that are excited by blue and near-infrared light, they can be designed to preferentially detect and synergically target and treat tumor cells commonly described as theranostic regimen [22,28].

### 4.1. Principle of Photodynamic Diagnosis and Photodynamic Therapy

**Figure 1:**
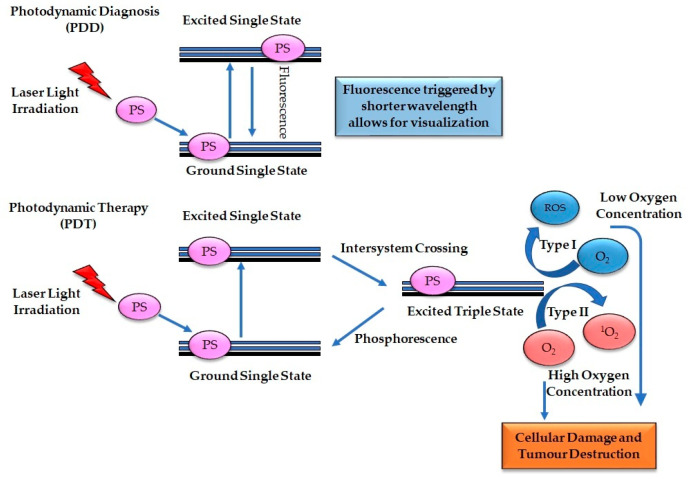
Principle of Photodynamic diagnosis and photodynamic therapy. Photoexcitation of a photosensitizer which stimulates fluorescence emission for cancer detection and a long-lived triplet state, which can trigger Type I or Type II cytotoxic oxygen species resulting in cell death.

The principle of PDD involves three steps: (a) internalization of photosensitizer in the tumor cells, (b) application of short wavelength of light (330 to 400 nm), and (c) emission of fluorescence. Therefore, the accumulation of the photosensitizer in the neoplastic tissue and the absorption of light results in the excitation of the photosensitizer from a ground state to a single excited state. The energy gain resulting from the change in status is expressed in the form of fluorescence (Figure 1), thereby facilitating diagnosis. The excitation generated is short-termed, and the photosensitizer cannot initiate any cellular processes; therefore, only diagnostic visualization can be achieved [28].

The principle of PDT also consists of three steps, however it varies from PDD: (a) internalization of photosensitizer in the tumor cells, (b) application of light, and (c) interaction with molecular oxygen. The mechanism begins with the accumulation of the photosensitizer into the cancer cells, and the application of light-based photosensitizer trigger the excitation of the photosensitizer from the single ground state to a single excited state. Furthermore, the conversion of the excited photosensitizer from singlet state to an excited triplet state via the intersystem crossing and the reaction with the neighboring molecular oxygen. This process produces type I or II cytotoxic oxygen species, leading to direct cancer cell death, damage of the tumor vasculature, and immunologic effects (Figure 1) [29].

Type I reaction involves the interaction of the electrons from the excited photosensitizer triplet state and the surrounding cellular components to produce superoxide reactive oxygen species (ROS), usually from cellular components with low molecular oxygen concentrations. At the same time, type II reaction involves the interaction of the electrons from the excited photosensitizer triplet state and high molecular oxygen from the adjacent cellular elements to generate singlet oxygen. Type II reaction is the most common PDT reaction pathway. Although both PDT reaction type I and type II depend on oxygen, the subcellular oxygen concentration within the tumor microenvironment determines the reaction type that occurs. The efficiency of PDT to induce tumor cell destruction relies on the concentration and subcellular internalization of the photosensitizers and the production of sufficient cytotoxic singlet molecular oxygen and ROS [30].

### 4.2. Characteristics of an Ideal Photosensitizer for Photodynamic Diagnosis and Photodynamic Therapy

A photosensitizer is a photochemical agent and a crucial component of the PDD and PDT processes. It is characterized by its selective retention in tumor cells and its activation by a specific wavelength of light to trigger fluorescence, phosphorescence, or cellular damage, thereby facilitating efficient PDD and PDT cancer treatment [31]. An excellent photosensitizer employed for PDD and PDT should fulfil the following criteria: (1) high affinity for cancer cells, (2) selective uptake and retention within tumor cells, (3) ability to fluorescence at the visible light spectrum and induction of cell death at the infrared or near-infrared light region, (4) efficient in producing ROS, (5) high chemical purity, homogenous in composition and amphiphilic, (6) stable in biological fluids, and (7) absence of dark cellular toxicity and rapidly remove from healthy tissues [31,32]. Photosensitizers are categorized in relation to their chemical configuration into; porphycenes, chlorins, porphyrins, and phthalocyanines. In addition, based on their historical development and specific functional modification, photosensitizers can be classified into first generation, second generation, and third generation (functionalized PS) [33].

### 4.3. Photosensitizer for Esophageal Cancer PDD Applications

The classic photosensitizer used in PDD of esophageal cancer consists of 5-aminolevulinic acid (5-ALA), indocyanine green, and methylene blue [23,24,25]. Among these photosensitizers, 5-ALA has attracted much attention as a promising agent for PDD in identifying cancer sites. 5-ALA is a metabolic precursor of protoporphyrin IX (PpIX). PPIX is a fluorescent by-product that releases a red fluorescence when blue light is excited at ~635 nm [34]. 5-ALA has been employed for PDD of various cancers such as bladder cancer [35], gastric cancer [36], and malignant glioma [37], as well as in esophageal cancer; this is due to its ability to fluorescence when activated within the visible blue and red light wavelength. It has minimal skin photosensitivity and absence of stricture [23,38,39,40].

Prompt diagnosis of lymph node metastasis is crucial in the treatment of cancer as well as survival rate. Despite the progress made in computed tomography and magnetic resonance imaging, smaller tumor nodules are not detected before surgery, and in the course of surgery, some are neglected [41,42]. The application of ALA-PDD in combination with the diagnostic laparoscopy/cystoscopy overcomes this problem and improves the rate of lymph node metastatic detection. A study by Motoori and coworkers used ALA-PDD to detect lymph node metastasis in patients with esophageal cancer. In this study, a total of 292 lymph nodes were examined from eight esophageal cancer patients following surgery. The outcome of ALA-PDD was compared with that of histopathological evaluation. The findings showed that out of the 292 lymph nodes, 21 nodes (7.2%) were positive for PDD, and 19 nodes (6.5%) were histologically positive, highlighting the effectiveness of this technique over standard histological evaluation [23].

### 4.4. Photosensitizer for Esophageal Cancer PDT Applications

Several PSs have been approved and are applied for the treatment of esophageal cancer [43,44]. First-generation PS have been well studied in PDT, and this includes the hematoporphyrins (Hp). The initial Hp was a combination of different porphyrins, with each having peculiar features. It was first employed as a fluorescent diagnostic tool for neoplastic lesions; however, high doses were required for optimal outcomes due to its diverse nature. Furthermore, it has very low tissue penetration depth and causes undesired photosensitivity reactions; this led to the development of porfimer sodium (Photofrin) [32]. Porfimer sodium is a purified hematoporphyrin derivative and a first-generation PS approved for clinical applications in PDD and PDT for the treatment of early-stage, superficial, and advanced staged esophageal cancer [43,45]. Studies have demonstrated an optimal complete response rate with porfimer sodium for superficial esophageal cancer and could be used for curative treatment [46,47,48]. However, porfimer sodium has several setbacks limiting its application, and these include inadequate cancer selectivity, insufficient absorption and penetration of light into the cancer cells to generate adequate reactive oxygen species as a result of its short wavelength absorption (630 nm) and severe skin phototoxicity [49,50,51]. The above limitations of the first-generation photosensitizers led to the development of second-generation photosensitizers.

Second-generation photosensitizers were designed with the following improved features over the first-generation photosensitizers: optimal tumor affinity, minimal side effects, improved light absorption and penetration with a longer wavelength within the infrared and near-infrared region (650–800 nm), and high chemical purity. The second-generation PSs are made up of various forms of porphyrins and are grouped into porphyrins, pheophorbides, chlorins, and phthalocyanines [32,52]. HPPH (2-[1-hexyloxyethyl]-2-devinyl pyropheophorbide-a), a prodrug of ALA (5-aminolevulinic acid), is a second-generation PS used for esophageal cancer with the advantage that it exhibits less photosensitization period. However, relapse may occur due to inadequate tumor penetration [53].

Chlorins are a modified version of porphyrins and a second-generation PS. The PSs under group used for esophageal cancer include m-tetrahydroxyphenyl chlorine (mTHPC, Foscan) and talaporfin sodium (mono-aspartyl chlorine e6). Foscan is a potent drug for high-grade dysplasia and early-stage esophageal cancer [54]. This drug is associated with stricture, tissue damage, skin photosensitivity for a long duration. Talaporfin sodium is an efficient curative and salvage therapy for esophageal cancer with local failure following chemoradiotherapy without metastases. It has no adverse effect and is shown to improve the overall survival rate and patient quality of life; however, it is suboptimal for more extensive tumors [18,19,49,55].

Phthalocyanine (Pc) is a second-generation macrocyclic PS and a derivative of porphyrins. Phthalocyanine has also been investigated for various esophageal cancer cell lines and has shown to be effective as an emerging photosensitizer for the treatment of esophageal cancer. These PSs have high absorption in the visible red-light spectrum and generate elevated ROS, crucial for effective cell death. A study with two metallophthalocyanine compounds—mixed-sulfonated aluminium phthalocyanine (AlPcSmix) and mixed-sulfonated germanium phthalocyanine (GePcSmix)—found that they were effective for SNO esophageal cancer cell line with dose-dependent cytotoxicity. In this study, the cell death impact was more effective at a lower concentration of photoactivated AlPcSmix and GePcSmix [56]. Another study by Kuzyniak et al. observed that when 10 μM of ZnPc PS was administered to in vitro cultured Kyse-140 (squamous cell carcinoma) and OE-33 (adenocarcinoma) of esophageal cancer cell lines, no photosensitivity was observed. In addition, activation at 633 nm light wavelength and a fluence of 10 J/cm^2^ resulted in >90% reduction of viable cells with significant cellular damages [57]. Other metallate PCs such as mixed-sulfonated tin phthalocyanine (SnPcSmix) and mixed-sulfonated silicon phthalocyanine (SiPcSmix) have likewise been investigated for esophageal cancer and demonstrated significant cell death with no adverse effects [58].

### 4.5. Limitations and Strategies to Improve Photodynamic Diagnosis and Photodynamic Therapy

There are no doubts that PDD and PDT are characterized with the following advantage over conventional cancer therapy. It is noninvasive; minimal systemic toxicity as the PS can only be activated when light is applied. It can selectively cause tumor cell death without affecting the normal tissue. It can be applied as curative therapy, neoadjuvant therapy, adjuvant therapy, or palliative therapy. Furthermore, it is less expensive when compared with the classic treatment [31]. However, PDT application in the clinical setting is limited due to the hydrophobic characteristics of the PSs, which can significantly hinder their solubility, causing systemic aggregation, preventing uniform distribution, reduced cellular accumulation and low tissue specificity of the standard PS agents, low ROS production, and skin photosensitivity. In addition, the application of PDD and PDT in deep solid tumors and distant metastasis is limited due to inadequate light penetration through tumor tissues. Therefore, to overcome the above limitations, different approaches have been employed, which include PDT combined with conventional therapy and targeted PDT.

### 4.6. Photodynamic Therapy in Combination with Conventional Therapy

Conventional therapy in combination with PDT offers a promising approach to improving the effectiveness of cancer treatment. The unique features of PDT, such as fewer side effects and extensive induction of anti-tumor immune responses, makes it favorable with other treatment approaches like surgery, radiotherapy or chemotherapy [59,60]. Surgery in combination with PDT has been well studied. This combination approach limits the possibility of bypassing any unnoticed minute cancer nests. Findings have shown a significant decrease in metastases, enhanced anti-tumor immune response, and rate of tumor regrowth [37,61]. The application of PDT with surgery can be performed as a neoadjuvant PDT [62], intraoperative PDT [63] and postoperative PDT [64]. Studies have reported that this combined measure improves patient treatment outcomes.

### 4.7. Targeted Photodynamic Diagnosis and Photodynamic Therapy

The quest to develop a more targeted PDT strategy resulted in the emergence of third-generation PSs. These new PS are characterized by high PS solubility, quick clearance from the body, effective biodistribution, efficient intercellular penetration and internalization into targeted tumor cells [32,33]. These third-generation PSs are improved versions of either first-generation or second-generation PSs, which are functionally modified, conjugated to carrier molecules such as nanoparticles, liposomes, and targeting moieties such as monosaccharides and peptides monoclonal antibodies (mAbs), and aptamers. The main goal of incorporating PSs to carrier molecules and targeting moieties is to boost tumor PS drug selective adsorption, cellular internalization, and thereby facilitating optimal theranostic outcome [31,33].

## 5. Photoimmunotherapy for Targeted Photodynamic Diagnosis and Photodynamic Therapy

Photo-immunotherapy is a recently developed cancer therapy that involves the conjugation of antibodies to a photosensitizer. This antibody-photosensitizer conjugate (APC) binds to specific cancer cells expressing the complementary antigen on the cell surface [65]. Applying light to the cancer tissue at an appropriate wavelength induces immunogenic cell death (ICD). The ICD stimulates the actions of the adaptive immune response against several tumor antigens released from the dying cancer cells. This action potentiates the therapeutic efficiency of PIT when compared with the conventional PDT [65,66,67,68]. In addition, PIT preferentially destroys target cancer cells expressing only the corresponding antigens, thereby sparing the normal cells, unlike PDT, which allows some photosensitizer accumulation in normal tissue. Among the several antibodies used in the PIT for esophageal cancer, antibodies targeting EGFR [69], HER2 [70] and cancer-associated fibroblasts (CAFs) have received much attention [71]. This is because these receptors/proteins are overexpressed in esophageal cancer cells.

In 2018, Pye et al. designed a photoimmunoconjugate for PDD and PDT of esophageal cancer. The photoimmunoconjugate comprises trastuzumab, a HER2-directed antibody with two photosensitizers consisting of a NIR-dye with fluorescence detection and chlorin e6, which generate ROS for cancer destruction. This photoimmunoconjugate allows for simultaneous diagnosis and treatment of esophageal cancer [70]. Furthermore, in another study, Hartman et al. designed targeted photoimunoconjugates made of cetuximab-IRDye700DX and trastuzumab-IRDye700DX for the tumor targeting the delivery of the PSs [69]. Cetuximab and trastuzumab are monoclonal antibodies of EGFR and HER2, respectively, which are overexpressed on many esophageal cancer cells. The Cetuximab and trastuzumab decoration promote cell recognition and internalization of the IRDye700DX during treatment, which accelerates the release of the PSs within the cancer cells. In addition, another study indicated that CAF targeted PIT could significantly halt the tumor growth of esophageal cancer and eliminate drug resistance associated with chemotherapy [71]. Therefore, PIT could be a potential therapeutic option for esophageal cancer.

## 6. Nanomedicine for Targeted Photodynamic Diagnosis and Photodynamic Therapy

**Figure 2:**
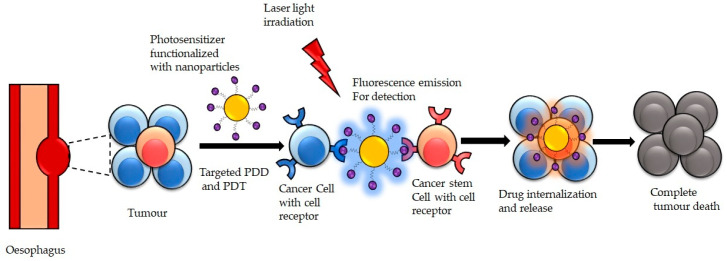
Nanoparticle interaction at the intracellular level facilitates cancer visualization, PS drug internalization, and tumor death.

PDT has experienced a revolution in the past years through the development of nanomedicine. These have led to improved controlled photoreaction, with sufficient fluorescence and ROS production. Furthermore, these have boosted tumor preferential specificity and the formulation of biomodulation approaches, increasing treatment efficiency [72,73], consequently enhancing the overall PDD and PDT outcomes. The emergence of nanotechnology and nanomedicine have made significant achievements in the early diagnosis and treatment of various diseases, including cancer. In medicine, nanotechnology involves the design and application of heterogeneous groups of small size particles with diameters ranging from 1 to 100 nm for disease diagnosis and treatment [74,75]. Cancer nanomedicine refers to the application of nanotechnology, leveraging its unique features to design novel measures that enable its interaction at the intracellular level for cancer diagnosis and treatment (Figure 2). Currently, several nanomedicine formulations have been employed to treat cancer [76].

Significant improvements in PDD and PDT have been made through the application of nanotechnology due to their abilities to overcome the various limitation of the conventional PS drug through effective distribution and delivery of the PS drug into the targeted cancer cells. The benefits of using nanotechnology such as nanoparticles (NPs) for PS drug delivery are characterized by their peculiar physicochemical attributes, ability to be chemically modified to attain selective and specific targeting, controlled release of the PS drugs, and improved treatment outcomes (Figure 2) [72,77]. In addition, NPs facilitate passive tumor PS drug uptake due to the permeability and retention (EPR) effect. The EPR passive uptake effects allow the free movement of NPPS drug into the tumor microvasculature leveraging on the porous blood vessels and the weak lymphatic drainage, thereby increasing the drug localization in the tumor cells while non-targeted healthy cells are spared. The small size of NPs promotes easy absorption by the tumor microvasculature, thereby promoting PS drug internalization. Furthermore, the large surface area to volume ratio of NPs allows a considerable amount of the PS drug load, consequently increasing the PS absorption concentration in the cancer cells. Furthermore, NPs shield PS drugs from being attacked by the body, owing to their ability to mimic biological molecules [72,78].

### 6.1. Nanomedicine Platform for Targeted Photodynamic Diagnosis and Photodynamic Therapy

For optimal and efficient targeted PDD and PDT, functionalized nanomedicine platforms have been designed to facilitate passive and active PS drug distribution in cancer cells. Presently, several nanomedicine platforms have been examined for targeted PS delivery, and these consist of organic, inorganic, and carbon-based NPs [78,79,80]. The application of these NP platforms/formulations in PDT has been proven as a beneficial diagnostic and therapeutic enhancement modality to boost tumor detection and cancer cell destruction [77].

Organic NPs are solid materials made of organic substances such as lipids, proteins, carbohydrates, or polymers. Examples of organic nanomedicine formulation for PDT include micelles, liposomes, dendrimers, ferritin, and polymers NPs (Table 1). Organic nanomaterials have unique features such as minimal toxicity, high solubility, which facilitate passive or active PS drugs localization within the target cancer cells, allowing for easy encapsulation with PS drug [77,78,80,81]. Inorganic NPs formulation comprises metal ions (e.g., Al, Cd, Co, Cu, Au, Fe, Pb, Ag, and Zn) and metal oxides (e.g., aluminium oxide (Al_2_O_3_), cerium oxide (CeO_2_), iron oxide (Fe_2_O_3_), magnetite (Fe_3_O_4_), silicon dioxide (SiO_2_), and titanium oxide (TiO_2_)) (Table 2). These NPs have distinctive attributes such as small sizes, large surface area to volume ratio, surface charge, high optical strength, multiple shapes, and structures. A potential strategy presented by inorganic-based NPs is that they increase drug payload and delivery of insoluble PS drug to cancer cells via the enhanced permeability and retention (EPR) effect. Furthermore, inorganic base NPs have a prolonged cycle period, slow clearance, and defined control release, which is beneficial for PDT. Moreover, they can be employed for diagnosis and imaging [77,79,80,81].

Carbon-based NPs have attracted much attention in the area of PDT. These NPs consist of carbon particles, and they have distinct biological and physiochemical characteristics that make an ideal candidate for PDT. These characteristics include high optical and mechanical attributes, high biocompatibility, minimal toxicity, extensive chemical modifications, improved enhanced permeability effect, heat generation and conductivity features, as well as enhanced ROS production [31,85]. Examples of carbon-based NPs include carbon nanotubes (CNT), carbon nanofibers, fullerenes (C60), and graphene (Table 3) [77,80]. Carbon nanotubes, especially single-walled carbon nanotubes, are highly effective nanocarriers for insoluble PS drugs. Therefore, making them a promising candidates for cancer treatment [86]. Xue et al. evaluated a novel natural biomass carbon dots (NBCDs) using the exocarp of lychee and functionally encapsulated transferrin and Ce6 on the surface to create a NIR fluorescence imaging nanoprobe. The findings showed that NBCD-PEG-Ce6-Tf nanoprobes emit NIR fluorescence and offer desirable biosafety. Furthermore, the Ce6 photosensitizer can generate high ROS through photodynamic activation upon light irradiation, resulting in cell death and preventing tumor proliferation in PDT-treated mice. Therefore, these NBCD-PEG-Ce6-Tf nanoprobes could be employed for PDD and PDT [87].

**Table 2:** Inorganic NP platforms and characteristics applied for targeted PDD and PDT.

Inorganic NP Platforms	Characteristics	References
Gold NPs	Allows for surface functionalization Excellent optical/photoresponsive feature Good biocompatibility Minimal toxicity High ROS production Enhance cancer cell destruction Improve drug delivery target site surface Plasmon resonance (LSPR) characteristics	[73,77,88,89]
Silver NPs	Brilliant optical and physiochemical features High production of ROS High surface volume to ratio Easy surface modification Enhance anti-tumor effects Excellent antimicrobial activity	[89,90]
Manganese oxide	High oxygen production potential Overcomes tumor hypoxia Improve anti-tumor effects High light absorption strength absorption ability Excellent biocompatibility	[91]
Titanium dioxide	Good photosensitive agent Ability to produce singlet oxygen Allows for bandgap and band position, Highly photostable Non-toxicity, Excellent catalytic activity, Highly abundance and affordability	[89,91]

### 6.2. Functionalized Nanomedicine for Targeted PDD and PDT of Esophageal Cancer

These above NPs platforms can be functionalized using unique tumor targeting surface receptors or ligand agents such as monoclonal antibodies (mAb), antibody fragments, aptamers and nucleic acid, small molecules, and peptides. These ligands are added to the surface of NPs, enabling the optimal coupling of the nanocarrier PS conjugates to overexpress cancer surface recognition molecules, such as epidermal growth factor receptors (EGFR), folate receptors (FR), transferrin receptors (TfR), CD44, and CD133, to improve PS subcellular internalization and accumulation [96,97,98]. Two main strategies are employed for efficient delivery and localization of functionalized NPPS drugs to the target cancer cells: active and passive nanotargeted drug delivery systems (Table 4). These systems depend on the biodistribution and the localization of the NPPS to boost PDD and PDT; however, for increased specificity for tumor cells, active targeting moieties coupled into NPs have been shown to facilitate the efficient accumulation of NPPS drugs.

#### Passive and Active Functionalized Nanomedicine for Targeted PDD and PDT of Esophageal Cancer

**Figure 3:**
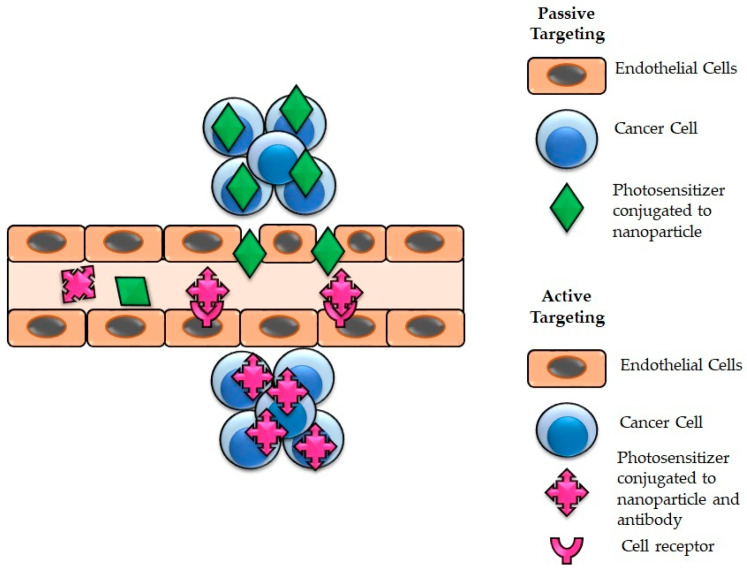
Enhanced cellular accumulation of PDT photosensitizer drugs using passive and active functionalized nanomedicine cancer targeting and delivery mechanisms.

Passive targeting strategy solely relies on the physicochemical properties of PS drugs/NPs and the pathophysiological characters of the cancer cells. It is hypothesized that the EPR effect, which takes advantage of the porous tumor endothelium, and the weak tumor lymphatic drainage, promote the selective localization of the PS drugs/NPs in cancer cells (Figure 3) [81,97,98]. Active targeting refers to the cellular and molecular tumor site interactions through surface antigens (Figure 3). Several biomolecules associated with cancer and cancer microenvironment as emerged as active targeting ligand agents for the functionalized surface of nanoplatforms, and these include monoclonal antibodies (mAb), aptamers, small molecules, peptides, and nucleic acids [81,96,97,98]. The conjugation to specific ligands or surface receptors in esophageal cancer allows targeted delivery of NPPS to esophageal cancer cells, thus significantly enhancing the PDD and PDT effectiveness of esophageal cancer treatment.

In 2016, Li et al. demonstrated an effective NIR visualization of early-stage esophageal squamous cell carcinoma model using a nanoprobe that consists of polyamidoamine (PAMAM) dendrimer labeled with a NIR dye Cy5.5 and conjugated with cyclic RGDfK peptide targeting the αvβ3 integrin overexpressed in esophageal cancer cells. The findings from this study showed that the targeted nanoprobe enhance and allowed for the detection of early-stage esophageal cancer [99]. Furthermore, to demonstrate NP-regulated controllable and sustained drug release for improved anti-tumor effectiveness in esophageal cancer cell lines, Wang and coworkers designed a NIR copper-based chalcogenide nanosystem containing core-shell silica materials (Cu_9_S_5_@MS-NIR). The result showed excellent tumor cell death, and the nanoformulation can be used as an efficient drug carrier [100].

A recent study reported the beneficial impact of fluoroscopy-assisted PDT combined with nanoparticle albumin-bound paclitaxel (Nab-P) in removing the tumor mass, keeping the organ intact without losing its function, and excellent treatment outcomes in locally advanced esophageal cancer [101]. To improve drug biodistribution, bioavailability, and efficient treatment, Li and coworkers use trimethyl chitosan (TMC) coated irinotecan (IRN) loaded solid lipid nanoparticle SLNs (TMC-IRN-SLNs) to treat esophageal cancer cells. This strategy showed increased drug internalization in the cancer cells and cancer cell death, thus improving the optimal treatment for esophageal cancer [102]. For effective targeted cancer drug release, detection, and treatment, Fan et al. developed a nanoconjugate for the treatment of esophageal cancer. The nanoconjugates consisting of fluorescent-peptide nanoparticles (f-PNPs) coupled to RGD (cyclic peptides) loaded with epirubicin (EPI) form the RGD-f-PNPs/EPI conjugates. The RGD preferentially bind to the αvβ3 integrin overexpressed in esophageal cancer cells. Findings from this study revealed efficient drug release, cancer detection and strong anti-cancer effect on esophageal cancer cells. Therefore, it could serve as an emerging modality for control drug release, cancer diagnosis and treatment [104].

To overcome tumor hypoxia and improve esophageal cancer detection, diagnosis, and treatment, a recent study by Liu et al. prepared and synthesized a carbon nanocages-based nanozyme system [103]. This system comprised of bovine serum albumin (BSA) manganese dioxide (MnO_2_) (BM), coupled on the surface of IR820 photosensitizer-assembled carboxylated carbon nanocages. The system showed increased drug load capacity, high drug release and localization into the cancer cell. Upon internalization, the tumor microenvironment activates the nanosystem, which in turn converts hydrogen peroxide into oxygen by their catalase-like activity to facilitate ROS generation of PDT. Under photoactivation, this promotes PDD visualization, PDT and photothermal effects through different photonic pathways for cancer detection and efficient esophageal cancer treatment [103].

### 6.3. Nano-Immunoconjugates for PDD and PDT of Esophageal Cancer

**Figure 4:**
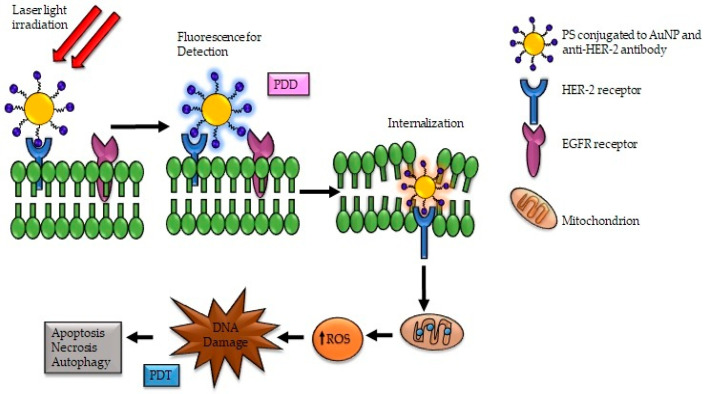
Antibody-based AuNP binding to the HER-2 receptor expressed in esophageal cancer cells promotes easy detection and diagnosis of esophageal cancer.

Immunoconjugates is the most prominent nanomedicine platform exploited for the active targeting PS delivery for esophageal cancer, with the anti-HER-2 and anti-EGFR antibodies being the most employed [69,70]. A recent study by Li and coworkers developed a sensitive and selective multifunctional AuNPs using resonance Rayleigh scattering assay to detect and diagnose esophageal cancer. The AuNPs was functionalized with anti-EGFR antibody and Anti-EGFR aptamer to detect esophageal cancer through the EGFR receptor selectively. The nanoprobe exhibited high sensitivity for esophageal cancer [105]. A similar study by Wang et al. demonstrated the applicability of antibody-based surface-enhanced Raman scattering (SERS) nanoparticles (NPs) to enhance esophageal cancer detection through endoscopic imaging to identify cell surface biomolecules [106]. These antibody-based SERS NPs target and bind to the EGFR and HER2, overexpressed in the cancer cells, thereby facilitating the rapid detection and diagnosis of esophageal cancer (Figure 4).

## 7. Conclusions

PDT and PDD are emerging modalities in the diagnosis and treatment of esophageal cancer. Several PDT strategies have been approved and promising outcomes have emerged from preclinical and clinical studies of PDT and PDD. The application of PDT for esophageal cancer has shown to be effective as curative therapy for superficial esophageal cancer and as salvage therapy following conventional therapeutic failure. Despite the significant success, these modalities are not fully applied in the clinical setting due to not being effective for deep-seated tumors and metastatic cancer. Shortcomings of the conventional PSs, which include low solubility, adverse side effects, and lack of tumor selectivity and specificity, have limited their application in clinical settings. Functionalized nanomedicine has proved to be highly effective in addressing these shortcomings through passive and active functionalized targeted PSs delivery for PDD and PDT, as previously stated. Several functionalized nanomedicines conjugated to PSs for esophageal cancer exist, with the most explored being the immunoconjugates using anti-EGFR and anti-HER-2 antibodies.

Findings have demonstrated that nanomedicine targeted PDD and PDT for esophageal cancer could improve early tumor detection, enhance treatment efficiency, eliminate cancer relapse, overcome treatment resistance, and overall improve patient survival rate. While most published papers that we reviewed emphasized PDT mainly, the potential application for PDD may also be evaluated if the photosensitizer in the functionalized nanomedicine enables fluorescence emission when excited with an appropriate wavelength of light. Future research should focus on smart double-action functionalized nanomedicines for PDD and PDT that can synergistically diagnose early-stage and treat esophageal cancer. Further studies are needed to examine the physicochemical, pharmacokinetic properties, and safety profiles of the nanomedicine to achieve optimal accumulation and PS internalization in the target tumor tissues. Though the attachment of the PSs on the surface of NPs can improve their biocompatibility, their possible toxic effects should be taken into account. Therefore, it is paramount that the above review on targeted PDD and PDT treatment for esophageal cancer and the role of functionalized nanomedicine be evaluated further in clinical trials to enable clinical translation.

## Figures and Tables

**Table 1:** Organic NP platforms and characteristics applied for targeted PDD and PDT.

Organic NP Platforms	Characteristics	Reference
Liposome	They are bilayer phospholipid systems High biocompatibility Nano drug carrier for both soluble and insoluble drugs Limits dark toxicity associated with conventional PSs Enhance cellular uptake	[73,77,82]
Micelle	Amphiphilic self-assembled structure Exist in different nanoforms Facilitate delivery of hydrophobic and hydrophilic PS drug Enhance both passive and active drug delivery Easy surface functionalization High stability in suspension and biocompatibility	[73,77]
Dendrimer	The monodisperse and highly branched structure Sustained drug release, High solubilization potential, Increase drug payload Enhance colloidal, biocompatibility, and shelf stability	[77,83]
Polymeric NPs	Easy to formulate and synthesize Exit in diverse structure Good biocompatibility Increase drug permeability into the target site Allow for surface modification Increase drug solubility Protection drugs from biodegradation An excellent delivery system for PS	[84]

**Table 3:** Carbon-based NP platforms and characteristics applied for targeted PDD and PDT.

Carbon-Based NP Platforms	Characteristics	References
Carbon nanotube	High photothermal conversion strength Tunable fluorescence Allow for surface engineering Efficient nanocarrier for insoluble PS drugs Suitable candidate for PDT cancer treatment Allows for covalent and non-covalent modification attachment of PS drug Enhance photocytotoxic effect	[86,92]
Fullerenes	Allows for extensive functionalization, Photochemistry potentials Ability to self-assemble into supramolecular fullerosomes Resistance to photobleaching Serve as drug nanocarrier to the nuclear pore complex and tumor vasculature	[93,94]
Graphene	Good photothermal property High singlet oxygen production Allow high drug payload Increase generation of ROS High Electrical thermal capacity Enhance cellular internalization Improve anti-tumor efficiency of PDT	[88,92,95]

**Table 4:** Functionalized nanomedicine for targeted PDD and PDT of esophageal cancer.

Nanoparticle Platform	PS/Drug	Ligand	Target	Outcome	Ref.
Dendrimer	NIR dye Cy5.5	Cyclic RGDfK peptide	αvβ3 integrin	Facilitate early detection of esophageal cancer	[99]
Core-shell silica materials (Cu_9_S_5_@MS)	NIR copper-based chalcogenide	-	-	Effective nanocarrier Excellent cancer cell death	[100]
Albumin	Paclitaxel	-	-	Enhance tumor elimination Organ preservation Excellent for locally advanced esophageal cancer	[101]
Solid lipid nanoparticle	Chitosan coated irinotecan			Increase drug internalization Promote tumor destruction	[102]
Manganese oxide/Carbon nanocages	IR800	Albumin	-	Increase drug payload Increase drug release and cellular uptake Increase ROS production Allows for visualization	[103]
Fluorescent-peptide nanoparticles	Epirubicin	RGD	αvβ3 integrin	Enhance drug release and internalization Promote tumor visualization	[104]
Gold nanoparticles		Antibody/aptamer	HER2/EGFR	Improve and facilitate rapid esophageal cancer detection Very sensitive technique	[105,106]

## Data Availability

Not applicable.

## References

[1] Ferlay J., Colombet M., Soerjomataram I., Parkin D.M., Piñeros M., Znaor A., Bray F. (2021). Cancer statistics for the year 2020: An overview. Int. J. Cancer.

[2] Smyth E.C., Lagergren J., Fitzgerald R.C., Lordick F., Shah M.A., Lagergren P., Cunningham D. (2017). Oesophageal cancer. Nature Rev. Dis. Prim..

[3] Then E.O., Lopez M., Saleem S., Gayam V., Sunkara T., Culliford A., Gaduputi V. (2020). Esophageal Cancer: An Updated Surveillance Epidemiology and End Results Database Analysis. World J. Oncol..

[4] Klingelhöfer D., Zhu Y., Braun M., Brüggmann D., Schöffel N., Groneberg D.A. (2019). A world map of esophagus cancer research: A critical accounting. J. Transl. Med..

[5] Spataro J., Zfass A.M., Schubert M., Shah T. (2019). Early Esophageal Cancer: A Gastroenterologist’s Disease. Dig. Dis. Sci..

[6] Thrumurthy S.G., Chaudry M.A., Thrumurthy S.S.D., Mughal M. (2019). Oesophageal cancer: Risks, prevention, and diagnosis. BMJ.

[7] Cummings D., Wong J., Palm R., Hoffe S., Almhanna K., Vignesh S. (2021). Epidemiology, Diagnosis, Staging and Multimodal Therapy of Esophageal and Gastric Tumors. Cancers.

[8] Rice T.W., Patil D.T., Blackstone E.H. (2017). 8th edition AJCC/UICC staging of cancers of the esophagus and esophagogastric junction: Application to clinical practice. Ann. Cardiothorac. Surg..

[9] D’Journo X.B., Thomas P.A. (2014). Current management of esophageal cancer. J. Thorac. Dis..

[10] Watanabe M., Otake R., Kozuki R., Toihata T., Takahashi K., Okamura A., Imamura Y. (2020). Recent progress in multidisciplinary treatment for patients with esophageal cancer. Surg. Today.

[11] Moaven O., Wang T.N. (2019). Combined Modality Therapy for Management of Esophageal Cancer: Current Approach Based on Experiences from East and West. Surg. Clin..

[12] NCCN (2021). NCCN Clinical Practice Guidelines in Oncology (NCCN Guidelines®) for Esophageal and Esophagogastric Junction Cancers.

[13] Ikeda G., Yamamoto S., Kato K. (2021). The safety of current treatment options for advanced esophageal cancer after first-line chemotherapy. Expert Opin. Drug Saf..

[14] Shah M.A., Bennouna J., Doi T., Shen L., Kato K., Adenis A., Mamon H.J., Moehler M., Fu X., Cho B.C. (2021). KEYNOTE-975 study design: A Phase III study of definitive chemoradiotherapy plus pembrolizumab in patients with esophageal carcinoma. Fut. Oncol..

[15] He S., Xu J., Liu X., Zhen Y. (2021). Advances and challenges in the treatment of esophageal cancer. Acta Pharm. Sin. B.

[16] Yura M., Koyanagi K., Hara A., Hayashi K., Tajima Y., Kaneko Y., Fujisaki H., Hirata A., Takano K., Hongo K. (2021). Unresectable esophageal cancer treated with multiple chemotherapies in combination with chemoradiotherapy: A case report. World J. Clin. Cases.

[17] Yagi K., Toriumi T., Aikou S., Yamashita H., Seto Y. (2021). Salvage treatment after definitive chemoradiotherapy for esophageal squamous cell carcinoma. Ann. Gastroenterol. Surg..

[18] Yano T., Kasai H., Horimatsu T., Yoshimura K., Teramukai S., Morita S., Tada H., Yamamoto Y., Kataoka H., Kakushima N. (2017). A multicenter phase II study of salvage photodynamic therapy using talaporfin sodium (ME2906) and a diode laser (PNL6405EPG) forlocal failure afterchemoradiotherapy or radiotherapy for esophageal cancer. Oncotarget.

[19] Ishida N., Osawa S., Miyazu T., Kaneko M., Tamura S., Tani S., Yamade M., Iwaizumi M., Hamaya Y., Furuta T. (2020). Photodynamic Therapy Using Talaporfin Sodium for Local Failure after Chemoradiotherapy or Radiotherapy for Esophageal Cancer: A Single Center Experience. J. Clin. Med..

[20] Hatogai K., Yano T., Kojima T., Onozawa M., Daiko H., Nomura S., Yoda Y., Doi T., Kaneko K., Ohtsu A. (2016). Salvage photodynamic therapy for local failure after chemoradiotherapy for esophageal squamous cell carcinoma. Gastrointest. Endosc..

[21] Koo M.M., Unger-Saldaña K., Mwaka A.D., Corbex M., Ginsburg O., Walter F.M., Calanzani N., Moodley J., Rubin G.P., Lyratzopoulos G. (2021). Conceptual Framework to Guide Early Diagnosis Programs for Symptomatic Cancer as Part of Global Cancer Control. JCO Glob. Oncol..

[22] Hu Y., Masamune K. (2017). Flexible laser endoscope for minimally invasive photodynamic diagnosis (PDD) and therapy (PDT) toward efficient tumor removal. Opt. Express.

[23] Motoori M., Yano M., Tanaka K., Kishi K., Takahashi H., Inoue M., Saito T., Sugimura K., Fujiwara Y., Ishikawa O. (2015). Intraoperative photodynamic diagnosis of lymph node metastasis in esophageal cancer patients using 5-aminolevulinic acid. Oncol. Lett..

[24] He J., Yang L., Yi W., Fan W., Wen Y., Miao X., Xiong L. (2017). Combination of Fluorescence-Guided Surgery With Photodynamic Therapy for the Treatment of Cancer. Mol. Imaging.

[25] Sasaki M., Tanaka M., Ichikawa H., Suzuki T., Nishie H., Ozeki K., Shimura T., Kubota E., Tanida S., Kataoka H. (2021). 5-aminolaevulinic acid (5-ALA) accumulates in GIST-T1 cells and photodynamic diagnosis using 5-ALA identifies gastrointestinal stromal tumors (GISTs) in xenograft tumor models. PLoS ONE.

[26] Agostinis P., Berg K., Cengel K.A., Foster T.H., Girotti A.W., Gollnick S.O., Hahn S.M., Hamblin M.R., Juzeniene A., Kessel D. (2011). Photodynamic therapy of cancer: An update. Cancer J. Clin..

[27] Kwiatkowski S., Knap B., Przystupski D., Saczko J., Kędzierska E., Knap-Czop K., Kotlińska J., Michel O., Kotowski K., Kulbacka J. (2018). Photodynamic therapy—Mechanisms, photosensitizers and combinations. Biomed. Pharmacother..

[28] Dobson J., de Queiroz G.F., Golding J.P. (2018). Photodynamic therapy and diagnosis: Principles and comparative aspects. Vet. J..

[29] Wang Y.-Y., Liu Y.-C., Sun H., Guo D.-S. (2019). Type I photodynamic therapy by organic–inorganic hybrid materials: From strategies to applications. Coord. Chem. Rev..

[30] Zhang Z.J., Wang K.P., Mo J.G., Xiong L., Wen Y. (2020). Photodynamic therapy regulates fate of cancer stem cells through reactive oxygen species. World J. Stem Cells.

[31] Niculescu A.-G., Grumezescu A.M. (2021). Photodynamic Therapy—An Up-to-Date Review. Appl. Sci..

[32] Abrahamse H., Hamblin M.R. (2016). New photosensitizers for photodynamic therapy. Biochem. J..

[33] Mfouo-Tynga I.S., Dias L.D., Inada N.M., Kurachi C. (2021). Features of third generation photosensitizers used in anticancer photodynamic therapy: Review. Photodiagn. Photodyn. Ther..

[34] Hinnen P., de Rooij F., Van Velthuysen M., Edixhoven A., Van Hillegersberg R., Tilanus H., Wilson J., Siersema P. (1998). Biochemical basis of 5-aminolaevulinic acid-induced protoporphyrin IX accumulation: A study in patients with (pre) malignant lesions of the oesophagus. Br. J. Cancer.

[35] Denzinger S., Burger M., Walter B., Knuechel R., Roessler W., Wieland W.F., Filbeck T. (2007). Clinically relevant reduction in risk of recurrence of superficial bladder cancer using 5-aminolevulinic acid-induced fluorescence diagnosis: 8-year results of prospective randomized study. Urology.

[36] Kishi K., Fujiwara Y., Yano M., Inoue M., Miyashiro I., Motoori M., Shingai T., Gotoh K., Takahashi H., Noura S. (2012). Staging laparoscopy using ALA-mediated photodynamic diagnosis improves the detection of peritoneal metastases in advanced gastric cancer. J. Surg. Oncol..

[37] Stummer W., Pichlmeier U., Meinel T., Wiestler O.D., Zanella F., Reulen H.-J., Group A.-G.S. (2006). Fluorescence-guided surgery with 5-aminolevulinic acid for resection of malignant glioma: A randomised controlled multicentre phase III trial. Lancet Oncol..

[38] Dunn J., Lovat L. (2008). Photodynamic therapy using 5-aminolaevulinic acid for the treatment of dysplasia in Barrett’s oesophagus. Expert Opin. Pharmacother..

[39] Mackenzie G.D., Dunn J.M., Selvasekar C.R., Mosse C.A., Thorpe S.M., Novelli M.R., Bown S.G., Lovat L.B. (2009). Optimal conditions for successful ablation of high-grade dysplasia in Barrett’s oesophagus using aminolaevulinic acid photodynamic therapy. Lasers Med. Sci..

[40] Casas A. (2020). Clinical uses of 5-aminolaevulinic acid in photodynamic treatment and photodetection of cancer: A review. Cancer Lett..

[41] Nishimaki T., Tanaka O., Ando N., Ide H., Watanabe H., Shinoda M., Takiyama W., Yamana H., Ishida K., Isono K. (1999). Evaluation of the accuracy of preoperative staging in thoracic esophageal cancer. Ann. Thorac. Surg..

[42] Wu L.F., Wang B.Z., Feng J.L., Cheng W.R., Liu G.R., Xu X.H., Zheng Z.C. (2003). Preoperative TN staging of esophageal cancer: Comparison of miniprobe ultrasonography, spiral CT and MRI. World J. Gastroenterol..

[43] Wu H., Minamide T., Yano T. (2019). Role of photodynamic therapy in the treatment of esophageal cancer. Dig. Endosc..

[44] Van Straten D., Mashayekhi V., De Bruijn H.S., Oliveira S., Robinson D.J. (2017). Oncologic Photodynamic Therapy: Basic Principles, Current Clinical Status and Future Directions. Cancers.

[45] Inoue T., Ishihara R. (2020). Photodynamic Therapy for Esophageal Cancer. Clin. Endosc..

[46] Craig C., Gray J., Macpherson M., Hodgson H., Zammit M., Fullarton G. (2007). Porfimer sodium photodynamic therapy in the treatment of early oesophageal carcinoma. Photodiagn. Photodyn. Ther..

[47] Nakamura T., Fukui H., Shirakawa K., Fujii Y., Fujimori T., Terano A. (2004). Photodynamic therapy of superficial esophageal cancer with a transparent hood. J. Gastrointest. Endosc..

[48] Tanaka T., Matono S., Nagano T., Murata K., Sueyoshi S., Yamana H., Shirouzu K., Fujita H. (2011). Photodynamic therapy for large superficial squamous cell carcinoma of the esophagus. Gastrointest. Endosc..

[49] Amanuma Y., Horimatsu T., Ohashi S., Tamaoki M., Muto M. (2021). Association of local complete response with prognosis after salvage photodynamic therapy for esophageal squamous cell carcinoma. Dig. Endosc..

[50] Mehraban N., Freeman H.S. (2015). Developments in PDT Sensitizers for Increased Selectivity and Singlet Oxygen Production. Materials.

[51] Ormond A.B., Freeman H.S. (2013). Dye Sensitizers for Photodynamic Therapy. Materials.

[52] O’Connor A.E., Gallagher W.M., Byrne A.T. (2009). Porphyrin and Nonporphyrin Photosensitizers in Oncology: Preclinical and Clinical Advances in Photodynamic Therapy. Photochem. Photobiol..

[53] Nava H.R., Allamaneni S.S., Dougherty T.J., Cooper M.T., Tan W., Wilding G., Henderson B.W. (2011). Photodynamic therapy (PDT) using HPPH for the treatment of precancerous lesions associated with Barrett’s esophagus. Lasers Surg. Med..

[54] Lovat L.B., Jamieson N.F., Novelli M.R., Mosse C.A., Selvasekar C., Mackenzie G.D., Thorpe S.M., Bown S.G. (2005). Photodynamic therapy with m-tetrahydroxyphenyl chlorin for high-grade dysplasia and early cancer in Barrett’s columnar lined esophagus. Gastrointest. Endosc..

[55] Hayashi T., Asahina Y., Nakanishi H., Terashima T., Okamoto K., Yamada S., Takatori H., Kitamura K., Mizukoshi E., Ninomiya I.J.E. (2020). Evaluation of the efficacy and safety of salvage photodynamic therapy by talaporfin sodium for cervical esophageal cancers and lesions larger than 3 cm. Esophagus.

[56] Kresfelder T.L., Cronjé M.J., Abrahamse H. (2009). The effects of two metallophthalocyanines on the viability and proliferation of an esophageal cancer cell line. Photomed. Laser Surg..

[57] Kuzyniak W., Schmidt J., Glac W., Berkholz J., Steinemann G., Hoffmann B., Ermilov E.A., Gürek A.G., Ahsen V., Nitzsche B. (2017). Novel zinc phthalocyanine as a promising photosensitizer for photodynamic treatment of esophageal cancer. Int. J. Oncol..

[58] Seotsanyana-Mokhosi I., Kresfelder T., Abrahamse H., Nyokong T. (2006). The effect of Ge, Si and Sn phthalocyanine photosensitizers on cell proliferation and viability of human oesophageal carcinoma cells. J. Photochem. Photobiol. B Biol..

[59] Firczuk M., Winiarska M., Szokalska A., Jodlowska M., Swiech M., Bojarczuk K., Salwa P., Nowis D. (2011). Approaches to improve photodynamic therapy of cancer. Front. Biosci..

[60] Gunaydin G., Gedik M.E., Ayan S. (2021). Photodynamic Therapy for the Treatment and Diagnosis of Cancer—A Review of the Current Clinical Status. Front. Chem..

[61] Kaibori M., Kosaka H., Matsui K., Ishizaki M., Matsushima H., Tsuda T., Hishikawa H., Okumura T., Sekimoto M. (2021). Near-Infrared Fluorescence Imaging and Photodynamic Therapy for Liver Tumors. Front. Oncol..

[62] Akopov A., Rusanov A., Gerasin A., Kazakov N., Urtenova M., Chistyakov I. (2014). Preoperarive endobronchial photodinamic therapy improves resectability in initially irresectable (inoperable) locally advanced non small cell lung cancer. Photodiagn. Photodyn. Ther..

[63] Muragaki Y., Akimoto J., Maruyama T. (2013). Phase II clinical study on intraoperative photodynamic therapy with talaporfin sodium and semiconductor laser in patients with malignant brain tumors. J. Neurosurg..

[64] Poorten V., Meulemans J., Nuyts S. (2015). Postoperative photodynamic therapy as a new adjuvant treatment after robot-assisted salvage surgery of recurrent squamous cell carcinoma of the base of tongue. World J. Surg. Oncol..

[65] Wakiyama H., Kato T., Furusawa A., Choyke P.L., Kobayashi H. (2021). Near infrared photoimmunotherapy of cancer; possible clinical applications. Nanophotonics.

[66] Turubanova V.D., Balalaeva I.V., Mishchenko T.A., Catanzaro E., Alzeibak R., Peskova N.N., Efimova I., Bachert C., Mitroshina E.V., Krysko O. (2019). Immunogenic cell death induced by a new photodynamic therapy based on photosens and photodithazine. J. Immunother. Cancer.

[67] Alzeibak R., Mishchenko T.A., Shilyagina N.Y., Balalaeva I.V., Vedunova M.V., Krysko D.V. (2021). Targeting immunogenic cancer cell death by photodynamic therapy: Past, present and future. J. Immunother. Cancer.

[68] Kobayashi H., Furusawa A., Rosenberg A., Choyke P.L. (2020). Near-infrared photoimmunotherapy of cancer: A new approach that kills cancer cells and enhances anti-cancer host immunity. Int. Immunol..

[69] Hartmans E., Linssen M.D., Sikkens C., Levens A., Witjes M.J., van Dam G.M., Nagengast W.B. (2017). Tyrosine kinase inhibitor induced growth factor receptor upregulation enhances the efficacy of near-infrared targeted photodynamic therapy in esophageal adenocarcinoma cell lines. Oncotarget.

[70] Pye H., Butt M.A., Funnell L., Reinert H.W., Puccio I., Rehman Khan S.U., Saouros S., Marklew J.S., Stamati I., Qurashi M. (2018). Using antibody directed phototherapy to target oesophageal adenocarcinoma with heterogeneous HER2 expression. Oncotarget.

[71] Katsube R., Noma K., Ohara T., Nishiwaki N., Kobayashi T., Komoto S., Sato H., Kashima H., Kato T., Kikuchi S. (2021). Fibroblast activation protein targeted near infrared photoimmunotherapy (NIR PIT) overcomes therapeutic resistance in human esophageal cancer. Sci. Rep..

[72] Alsaab H.O., Alghamdi M.S., Alotaibi A.S., Alzhrani R., Alwuthaynani F., Althobaiti Y.S., Almalki A.H., Sau S., Iyer A.K. (2020). Progress in Clinical Trials of Photodynamic Therapy for Solid Tumors and the Role of Nanomedicine. Cancers.

[73] Escudero A., Carrillo-Carrión C., Castillejos M.C., Romero-Ben E., Rosales-Barrios C., Khiar N. (2021). Photodynamic therapy: Photosensitizers and nanostructures. Mater. Chem. Front..

[74] Singh B., Mitragotri S. (2020). Harnessing cells to deliver nanoparticle drugs to treat cancer. Biotechnol. Adv..

[75] Zheng Y., Li Z., Chen H., Gao Y. (2020). Nanoparticle-based drug delivery systems for controllable photodynamic cancer therapy. Eur. J. Pharm. Sci..

[76] Raj S., Khurana S., Choudhari R., Kesari K.K., Kamal M.A., Garg N., Ruokolainen J., Das B.C., Kumar D. (2021). Specific targeting cancer cells with nanoparticles and drug delivery in cancer therapy. Semin. Cancer Biol..

[77] Gao D., Guo X., Zhang X., Chen S., Wang Y., Chen T., Huang G., Gao Y., Tian Z., Yang Z. (2020). Multifunctional phototheranostic nanomedicine for cancer imaging and treatment. Mater. Today Bio.

[78] Montaseri H., Kruger C.A., Abrahamse H. (2020). Review: Organic nanoparticle based active targeting for photodynamic therapy treatment of breast cancer cells. Oncotarget.

[79] Montaseri H., Kruger C.A., Abrahamse H. (2021). Inorganic Nanoparticles Applied for Active Targeted Photodynamic Therapy of Breast Cancer. Pharmaceutics.

[80] Sahu T., Ratre Y.K., Chauhan S., Bhaskar L.V.K.S., Nair M.P., Verma H.K. (2021). Nanotechnology based drug delivery system: Current strategies and emerging therapeutic potential for medical science. J. Drug Deliv. Sci. Technol..

[81] Kruger C.A., Abrahamse H. (2018). Utilisation of targeted nanoparticle photosensitiser drug delivery systems for the enhancement of photodynamic therapy. Molecules.

[82] Fahmy S.A., Azzazy H.M.E., Schaefer J. (2021). Liposome Photosensitizer Formulations for Effective Cancer Photodynamic Therapy. Pharmaceutics.

[83] Prajapati S.K., Maurya S.D., Das M.K., Tilak V.K., Verma K.K., Dhakar R.C. (2016). Dendrimers in drug delivery, diagnosis and therapy: Basics and potential applications. J. Drug Deliv. Ther..

[84] Sztandera K., Gorzkiewicz M., Klajnert-Maculewicz B. (2020). Nanocarriers in photodynamic therapy-in vitro and in vivo studies. Wiley Interdiscip. Rev. Nanomed. Nanobiotechnol..

[85] Chen J., Fan T., Xie Z., Zeng Q., Xue P., Zheng T., Chen Y., Luo X., Zhang H. (2020). Advances in nanomaterials for photodynamic therapy applications: Status and challenges. Biomaterials.

[86] Lu D., Tao R., Wang Z. (2019). Carbon-based materials for photodynamic therapy: A mini-review. Front. Chem. Sci. Eng..

[87] Xue M., Zhao J., Zhan Z., Zhao S., Lan C., Ye F., Liang H. (2018). Dual functionalized natural biomass carbon dots from lychee exocarp for cancer cell targetable near-infrared fluorescence imaging and photodynamic therapy. Nanoscale.

[88] Sun J., Kormakov S., Liu Y., Huang Y., Wu D., Yang Z. (2018). Recent Progress in Metal-Based Nanoparticles Mediated Photodynamic Therapy. Molecules.

[89] Mauro N., Utzeri M.A., Varvarà P., Cavallaro G. (2021). Functionalization of Metal and Carbon Nanoparticles with Potential in Cancer Theranostics. Molecules.

[90] El-Hussein A. (2016). Study DNA damage after photodynamic therapy using silver nanoparticles with A549 cell line. J. Nanomed. Nanotechnol..

[91] Yan K., Zhang Y., Mu C., Xu Q., Jing X., Wang D., Dang D., Meng L., Ma J. (2020). Versatile Nanoplatforms with enhanced Photodynamic Therapy: Designs and Applications. Theranostics.

[92] Hong E.J., Choi D.G., Shim M.S. (2016). Targeted and effective photodynamic therapy for cancer using functionalized nanomaterials. Acta Pharm. Sin. B.

[93] Bara’nska E., Wieche´c-Cudak O., Rak M., Bienia A., Mrozek-Wilczkiewicz A., Krzykawska-Serda M., Serda M. (2021). Interactions of a Water-Soluble Glycofullerene with Glucose Transporter 1. Analysis of the Cellular Effects on a Pancreatic Tumor Model. Nanomaterials.

[94] Hamblin M.R. (2018). Fullerenes as photosensitizers in photodynamic therapy: Pros and cons. Photochem. Photobiol. Sci..

[95] Monroe J.D., Belekov E., Er A.O., Smith M.E. (2019). Anticancer Photodynamic Therapy Properties of Sulfur-Doped Graphene Quantum Dot and Methylene Blue Preparations in MCF-7 Breast Cancer Cell Culture. Photochem. Photobiol..

[96] Fernandes S.R.G., Fernandes R., Sarmento B., Pereira P.M.R., Tomé J.P.C. (2019). Photoimmunoconjugates: Novel synthetic strategies to target and treat cancer by photodynamic therapy. Org. Biomol. Chem..

[97] Gierlich P., Mata A.I., Donohoe C., Brito R.M.M., Senge M.O., Gomes-da-Silva L.C. (2020). Ligand-Targeted Delivery of Photosensitizers for Cancer Treatment. Molecules.

[98] Gomez S., Tsung A., Hu Z. (2020). Current Targets and Bioconjugation Strategies in Photodynamic Diagnosis and Therapy of Cancer. Molecules.

[99] Li Q., Gu W., Liu K., Xiao N., Zhang J., Shao L., Li L., Zhang S., Li P. (2016). RGD conjugated, Cy5.5 labeled polyamidoamine dendrimers for targeted near-infrared fluorescence imaging of esophageal squamous cell carcinoma. RSC Adv..

[100] Wang S., Liu J., Qiu S., Yu J. (2019). Facile fabrication of Cu9-S5 loaded core-shell nanoparticles for near infrared radiation mediated tumor therapeutic strategy in human esophageal squamous carcinoma cells nursing care of esophageal cancer patients. J. Photochem. Photobiol. B Biol..

[101] Zhao W., Zhao J., Kang L., Li C., Xu Z., Li J., Zhang M. (2021). Fluoroscopy-Guided Salvage Photodynamic Therapy Combined with Nanoparticle Albumin-Bound Paclitaxel for Locally Advanced Esophageal Cancer after Chemoradiotherapy: A Case Report and Literature Review. Cancer Biotherapy Radiopharm..

[102] Ji C., Ju S., Zhang D., Qiang J. (2018). Nanomedicine Based N-Trimethyl Chitosan Entangled Solid Lipid Nanoparticle Loaded with Irinotecan to Enhance the Therapeutic Efficacy in Esophageal Cancer Cells. J. Biomater. Tissue Eng..

[103] Liu J., Gao J., Zhang A., Guo Y., Fan S., He Y., Yang K., Wang J., Cui D., Cheng Y. (2020). Carbon nanocage-based nanozyme as an endogenous H_2_O_2_-activated oxygenerator for real-time bimodal imaging and enhanced phototherapy of esophageal cancer. Nanoscale.

[104] Fan Z., Chang Y., Cui C., Sun L., Wang D.H., Pan Z., Zhang M. (2018). Near infrared fluorescent peptide nanoparticles for enhancing esophageal cancer therapeutic efficacy. Nat. Commun..

[105] Li J., Wang S., Kang W., Li N., Guo F., Chang H., Wei W. (2021). Multifunctional gold nanoparticle based selective detection of esophageal squamous cell carcinoma cells using resonance Rayleigh scattering assay. Microchem. J..

[106] Wang Y.W., Kang S., Khan A., Bao P.Q., Liu J.T.C. (2015). In vivo multiplexed Mol. Imaging of esophageal cancer via spectral endoscopy of topically applied SERS nanoparticles. Biomed. Opt. Express.

